# Targeting Cardiac Fibroblast Plasticity for Antifibrotic and Regenerative Therapy in Heart Failure

**DOI:** 10.3390/cells15020112

**Published:** 2026-01-08

**Authors:** Suchandrima Dutta, Sophie Chen, Waqas Ahmad, Wei Huang, Jialiang Liang, Yigang Wang

**Affiliations:** 1 Department of Pathology and Laboratory Medicine, College of Medicine, University of Cincinnati, 231 Albert Sabin Way, Cincinati, OH 45267, USA; duttasm@mail.uc.edu (S.D.); chen3sp@mail.uc.edu (S.C.); ahmadws@ucmail.uc.edu (W.A.); 2 Department of Internal Medicine, College of Medicine, University of Cincinnati, Cincinnati, OH 45267, USA; huangwe@ucmail.uc.edu

**Keywords:** fibroblast heterogeneity, fibrosis, single-cell transcriptomics, direct fibroblast reprogramming, antifibrotic therapy

## Abstract

**Highlights:**

**What are the main findings?**

- Cardiac fibroblasts (CFs) exhibit dynamic, state-dependent plasticity revealed by single-cell and spatial transcriptomics, with distinct subsets driving reparative versus maladaptive fibrotic remodeling in heart failure (HF).
- Fibroblast activation is regulated by coordinated signaling, mechanical, and epigenetic programs that stabilize chronic fibrosis but retain partial reversibility under defined conditions.

**What are the implications of the main findings?**

- Precision targeting of pathogenic fibroblast states, rather than global fibroblast suppression, offers a strategy to limit fibrosis while preserving essential reparative functions.
- Combining antifibrotic pathway modulation with in vivo fibroblast reprogramming, epigenetic editing, and advanced RNA/gene delivery platforms may enable reversal of established fibrosis and promote functional myocardial regeneration.

**Abstract:**

Cardiac fibrosis is a major component of heart failure (HF) and develops when reparative wound healing becomes chronic, leading to excessive extracellular matrix accumulation. Cardiac fibroblasts (CFs), the main regulators of matrix remodeling, are heterogeneous in developmental origins, regional localizations, and activation states. This diversity determines whether tissue repair resolves normally or progresses into maladaptive scarring that disrupts myocardial structure and function after injuries. Recent single-cell and spatial transcriptomic studies show that CFs exist in distinct yet interrelated molecular states in murine models and human cardiac tissue with specialized roles in matrix production, angiogenesis, immune signaling, and mechanical sensing. These insights redefine cardiac fibrosis as a dynamic and context-dependent process rather than a uniform cellular response. Although CFs are promising targets for preventing HF progression and enhancing cardiac remodeling, translation into effective therapies remains limited by the unclear heterogeneity of pathological fibroblasts, the lack of distinctive CF markers, and the broad activity of fibrogenic signaling pathways. In this review, we discuss the dynamics of CF activations during the development and progression of HF and assess the underlying pathways and mechanisms contributing to cardiac dysfunction. Additionally, we highlight the potential of targeting CFs for developing therapeutic strategies. These include nonspecific suppression of fibroblast activity and targeted modulation of the signaling pathways and cell populations that sustain chronic remodeling. Furthermore, we assess regenerative approaches that can reprogram fibroblasts or modulate their paracrine functions to restore functional myocardium. Integrating antifibrotic and regenerative strategies with advances in precision drug discovery and gene delivery offers a path toward reversing established fibrosis and achieving recovery in HF.

## 1. Introduction

Ischemic injury, most commonly resulting from myocardial infarction (MI), is a leading cause of cardiac dysfunction and heart failure (HF) [1]. The abrupt loss of blood flow deprives cardiomyocytes (CMs) of oxygen and nutrients, causing rapid, irreversible cell death [1,2]. Although early reperfusion therapy can limit the extent of necrosis, the majority of lost CMs are not replaced because the adult human heart possesses minimal regenerative capacity [2]. Instead, the damaged region undergoes a wound-healing process dominated by fibroblast activation and extracellular matrix (ECM) deposition [3,4]. The resulting fibrotic scar preserves structural integrity and prevents ventricular rupture but permanently alters tissue mechanics and electrical conduction, driving progressive ventricular remodeling that culminates in HF [4]. Thus, the limited regenerative potential of the heart, combined with sustained fibrotic remodeling, defines the chronic pathology that follows MI.

Cardiac fibroblasts (CFs) are the principal effector cells in this process. Under normal conditions, they maintain ECM turnover and contribute to the structural and mechanical stability of the myocardium [4,5]. After injury, quiescent fibroblasts transition into activated myofibroblasts that proliferate, secrete collagens, and remodel the ECM to reinforce the infarcted region [3,4]. While this activation is essential for early repair, its persistence leads to excessive matrix deposition, collagen crosslinking, and disorganization of myocardial structure [3,4]. These changes disrupt electrical coupling between surviving CMs, increase tissue stiffness, and compromise ventricular performance [4]. Cardiac fibrosis, therefore, represents a protective response that becomes maladaptive when unresolved.

While CFs hold potential as therapeutic targets to prevent HF progression, existing HF treatments continue to face substantial limitations. Existing pharmacologic therapies such as β-blockers, angiotensin-converting enzyme (ACE) inhibitors, and mineralocorticoid receptor antagonists mitigate fibrotic remodeling indirectly through neurohormonal inhibition but do not alter established scar tissue [6]. Some of these agents exhibit secondary antifibrotic effects by dampening fibroblast activation and collagen synthesis, yet they do not directly target the molecular pathways that sustain fibrosis [6]. Experimental interventions have focused on modulating key profibrotic signaling cascades, particularly transforming growth factor-β (TGF-β)/SMAD and connective tissue growth factor (CTGF) pathways, which regulate ECM production and myofibroblast persistence [3,7]. These strategies can reduce further matrix deposition and partially limit scar expansion but primarily stabilize existing fibrosis rather than restore lost myocardium.

Efforts to develop therapies targeting CFs are complicated by the molecular and functional similarity among fibroblasts across organs. Canonical structural markers such as collagen type I alpha 1 chain (*COL1A1*), decorin (*DCN*), and lumican (*LUM*) are widely expressed across multiple organs, while genes once considered cardiac-specific—periostin (*POSTN*) and collagen triple helix repeat-containing 1 (*CTHRC1*)—participate in conserved fibrotic programs shared with other tissues [8]. Nonspecific inhibition of fibroblast activation risks impairing normal wound healing or causing systemic effects. Single-cell and spatial transcriptomic studies in mouse hearts and increasingly in human failing myocardium have begun to resolve this complexity, identifying fibroblast subpopulations that change dynamically with time and region [8]. Early fibroblast states expressing transcription factor 21 (*TCF21*) and C-X-C motif chemokine ligand 12 (*CXCL12*), as defined in murine injury models, promote angiogenesis and scar stabilization, whereas late-stage *POSTN*^+^ and lysyl oxidase (*LOX*)^+^ fibroblasts reinforce stiffness and maladaptive remodeling [9]. These insights emphasize the need to selectively target fibroblast subsets responsible for chronic fibrosis while preserving transient reparative populations.

In addition to directly targeting pro-fibrogenic pathways or depleting pathological cell types, harnessing fibroblast plasticity emerges as an alternative strategy for reversing cardiac fibrosis [4]. The recognition that fibroblasts are diverse, plastic, and context-dependent has expanded their relevance from passive structural cells to active determinants of cardiac repair [4,9]. Advances in gene delivery and in vivo reprogramming, demonstrated primarily in murine models, now enable precise manipulation of fibroblast fate and activity [10]. Such strategies include direct conversion of fibroblasts into CM- or vascular-like cells, manipulation of fibroblast paracrine activity, and modification of the fibrotic microenvironment to support tissue renewal [10]. This review summarizes current understanding of fibroblast heterogeneity and activation in cardiac injury, examines antifibrotic and regenerative strategies under development, and discusses how these approaches may converge to achieve functional cardiac repair. While many mechanistic insights into fibroblast lineage dynamics, activation states, targeted ablation, and in vivo reprogramming have been derived from murine models, single-cell and single-nucleus transcriptomic analyses of injured human hearts have validated the presence of subsets of fibroblast populations and marker expression. Together, these methods define complementary goals: (1) antifibrotic therapy preserves myocardial structure by restraining pathological matrix remodeling and (2) regenerative therapy restores function by rebuilding lost tissue. Achieving both outcomes remains essential for durable cardiac recovery.

## 2. Fibroblast Heterogeneity and Function

Cardiac fibrosis contributes substantially to the structural and functional deterioration seen in HF, but its mechanisms cannot be understood without accounting for fibroblast diversity [3,4,5]. CFs constitute a heterogeneous population of mesenchymal cells that vary in developmental origin, spatial localization, and activation state [4,5]. This diversity determines how the myocardium responds to injury, whether repair remains controlled and reparative or transitions into maladaptive scar formation [3]. Recognizing and defining these distinctions is essential for understanding disease progression and designing therapies that modulate fibroblast behavior with precision.

Recent advances in single-cell and spatial transcriptomic profiling in human and murine tissue have transformed the understanding of fibroblast biology by revealing that these cells exist across a spectrum of molecular and functional states influenced by lineage history, microenvironment, and time after injury [8,9]. Distinct fibroblast subsets exhibit specialized functional signatures associated with ECM synthesis, angiogenesis, immune signaling, or mechanical sensing, underscoring that fibrosis arises from coordinated yet context-specific cellular processes rather than uniform activation [9]. These insights establish fibroblast heterogeneity as a key determinant of cardiac repair outcomes and a foundation for selective therapeutic targeting. The following subsections examine how developmental origins, molecular diversity, and temporal dynamics define CF function and how these features can be leveraged for antifibrotic and regenerative therapy development.

### 2.1. Developmental Origin and Regional Heterogeneity of Cardiac Fibroblasts

CFs originate primarily from mesodermal lineages and represent the main stromal population responsible for ECM integrity within the myocardium [5,11]. Lineage-tracing studies in murine models and single-cell analyses in both murine and human hearts demonstrate that adult CFs arise from multiple embryonic sources rather than a single progenitor pool [11,12]. Most ventricular CFs derive from epicardial cells through epithelial-to-mesenchymal transition (EMT) as shown in murine lineage-tracing studies, a process regulated by transcription factors such as *TCF21* that are essential for fibroblast specification and long-term maintenance [13,14]. A smaller fraction originates from endocardial endothelium via endothelial-to-mesenchymal transition (EndoMT), contributing mainly to fibroblast populations in the interventricular septum and atrioventricular junction [14,15]. These endocardial-derived CFs express genes related to valve morphogenesis and matrix remodeling, indicating region-specific specialization [5,14]. Additional contributions from neural crest-derived mesenchyme, as identified in murine developmental studies, occur in the outflow tract and great vessels, where they may influence vascular remodeling [11,16]. These embryonic lineages confer distinct transcriptional and epigenetic profiles that persist into adulthood, reflected in the expression of markers such as *TCF21*, *POSTN*, and platelet-derived growth factor receptor alpha (PDGFRα), and in differential sensitivity to TGF-β and WNT signaling, which shape lineage-specific activation thresholds [5,14,17].

While developmental origin defines foundational differences, spatial context further diversifies fibroblast states within the adult heart [14]. Comparative single-cell and spatial transcriptomic studies in murine model and human samples reveal that regional mechanical load and paracrine signaling drive distinct functional signatures [7,9]. Ventricular fibroblasts show enhanced integrin- and Hippo pathway activity, consistent with higher wall stress, whereas atrial fibroblasts display greater oxidative metabolism and secretory function [18,19]. Perivascular progenitor-derived fibroblasts express angiogenic factors contributing to vascular maintenance and, upon injury, support angiogenesis and scar remodeling [17,20]. Under physiological conditions, these distinctions remain stable, but following injury, they diverge further: ventricular fibroblasts expand robustly under pressure overload, while peri-infarct fibroblasts acquire inflammatory and matrifibrotic states that sustain scar maturation [9,17,21]. Together, these observations indicate that fibroblast heterogeneity reflects both developmental origin and continuous adaptation to the local biomechanical and biochemical environment.

Developmental and regional heterogeneity have direct implications for therapeutic targeting [5,17]. Broad expression of structural ECM genes across fibroblast subsets complicates selective manipulation, while context-dependent activation patterns restrict a temporal window for intervention [7,8]. Nevertheless, lineage- and region-restricted gene networks offer potential routes for precision delivery [11,13]. For example, promoters active predominantly in epicardial-derived or stress-responsive ventricular fibroblasts could enable localized targeted therapies directed to diseased myocardium while sparing quiescent fibroblasts elsewhere. Therefore, integrating lineage tracing, spatial transcriptomics, and chromatin accessibility data will be key to designing antifibrotic and regenerative strategies that exploit fibroblast diversity rather than attempting to suppress it globally.

### 2.2. Molecular Mechanisms of Fibroblast Activation

**Figure 1:**
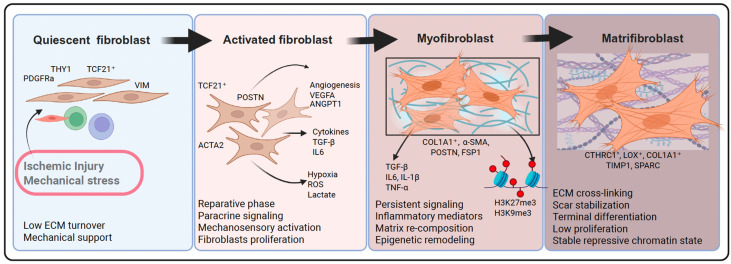
Sequential activation and stabilization of CFs during post-injury remodeling. Schematic representation of fibroblast state transitions following myocardial injury. Quiescent *PDGFRα*^+^/*THY1*^+^ fibroblasts activate in response to ischemic and mechanical stress, acquiring *TCF21*^+^/*POSTN*^+^/*ACTA2*^+^ phenotypes that mediate angiogenesis and paracrine repair. Sustained TGF-β and mechanical signaling drive differentiation into *COL1A1*^+^ myofibroblasts, which subsequently mature into *LOX*^+^/tissue inhibitor of metalloproteinases-1 (*TIMP1*)^+^ matrifibroblasts responsible for ECM crosslinking, scar stabilization, and establishment of a stable repressive chromatin state.

Resident CFs are the principal cellular contributors to cardiac remodeling following MI or other cardiac injuries, undergoing morphological and functional changes to adapt to the altered microenvironment (Figure 1). In the healthy myocardium, CFs remain in a quiescent state that maintains ECM turnover, preserves tissue compliance, and supports CM function through mechanical and paracrine signaling [4,14]. Quiescent CFs express canonical stromal markers such as PDGFRα, vimentin (VIM), and *THY1* but lack contractile proteins, including alpha smooth muscle actin (α-SMA/*ACTA2*) and *POSTN* [5,14,16]. Following myocardial injury, these cells rapidly activate in response to inflammatory and mechanical cues, entering a transient reparative phase that is essential for tissue stabilization [7,9,22]. Early-activated fibroblasts secrete angiogenic and immunomodulatory mediators such as vascular endothelial growth factor A (VEGFA), angiopoietin-1 (ANGPT1), and interleukin-6 (IL-6), which promote macrophage recruitment, endothelial proliferation, and CM survival [17,21]. Spatial transcriptomic datasets in murine MI models show that these populations localize to the infarct border zone, forming temporary signaling niches that coordinate scar formation and neovascularization [9]. At this stage, chromatin accessibility and DNA methylation profiles remain relatively permissive, with open enhancers at *POSTN* and *COL1A2* loci and preserved availability at cardiogenic genes such as *TBX20* and *GATA4* [11,23,24]. Single-cell trajectory analyses in human samples identify intermediate clusters co-expressing quiescent (PDGFRα, *TCF21*) and early-activation (*POSTN*, *ACTA2*) genes, suggesting that early activation involves stepwise transcriptional and chromatin changes that remain reversible under appropriate conditions [9,22,25].

When injury signals persist, sustained biomechanical and biochemical stimulation can stabilize the myofibroblast phenotype. TGF-β signaling acts as the principal inducer of this transition through SMAD2/3-dependent transcription of fibrotic genes, while CTGF amplifies ECM synthesis downstream [7,26]. Simultaneously, increased matrix stiffness and mechanical strain activate integrin-mediated pathways and the YAP/TAZ branch of Hippo signaling, driving a self-sustaining activation loop [18,27]. Persistent inflammatory mediators such as interleukin-1β (IL-1β) and tumor necrosis factor alpha (TNF-α) further modify fibroblast function, enhancing proliferation and altering matrix turnover [3,28]. Chronic exposure to these stimuli promotes metabolic reprogramming toward glycolysis and yields stable matrifibroblast subsets characterized by *CTHRC1*^+^, *COL1A1*^+^, and *POSTN*^+^ expression [7,25,29,30]. Single-cell trajectory analyses from human samples with ischemic cardiomyopathy delineate this temporal progression from early CF populations to late activated states, reflecting a transcriptional shift from reparative to pathogenic gene programs [31]. Multi-omic studies reveal widespread enhancer reorganization: TGF-β signaling recruits SMAD2/3 to *POSTN*, *LOXL2*, and *COL1A2* regions while closing cardiogenic enhancers near *TBX20* and *GATA4* [23,24,32]. Repressive histone modifications such as histone H3 lysine 27 trimethylation (H3K27me3) and DNA hypermethylation generate an “epigenetic scar” that maintains activation even after the initial stimuli subside, explaining the persistence of fibrosis once the acute phase has resolved [33,34].

Spatial and single-cell analyses from murine samples show that activation trajectories vary across the myocardium during the remodeling process [9,22,35]. Peri-infarct and border-zone fibroblasts express higher *POSTN* and *CTHRC1* levels, consistent with ongoing matrix synthesis, whereas fibroblasts in remote myocardium retain *TCF21* and *DCN* signatures indicative of quiescence [9]. Regional metabolic differences parallel these transcriptional states, with glycolysis dominating in actively remodeling zones and oxidative metabolism in uninjured regions [33]. Collectively, these observations define fibroblast activation as a dynamic, spatially patterned, and partially reversible process driven by cytokine, mechanical, and epigenetic inputs. Rather than existing as discrete quiescent and activated phenotypes, fibroblasts transition through a spectrum of states whose stability depends on the surrounding microenvironment and chromatin landscape [9,22,23]. Understanding the mechanisms that stabilize or reverse these states provides a foundation for therapies aimed at limiting pathological activation or reprogramming fibroblasts toward regenerative fates.

### 2.3. Roles of CFs Contributing to HF: Cell-Cell or Cell-ECM Interactions

Recent single-cell and spatial transcriptomic studies in murine models have clarified how specific fibroblast subsets contribute to the transition from adaptive repair to HF [9,21]. Late-stage *CTHRC1*^+^ and *LOX*^+^ fibroblasts generate a densely crosslinked ECM that stabilizes the infarct but reduces myocardial elasticity [25,30,36]. The resulting mechanical constraint limits diastolic relaxation and contributes to ventricular stiffening [4,5]. Additionally, regional heterogeneity of fibroblast activation can modify myocyte-fibroblast interactions and tissue conductivity [37]. Pathological fibroblasts also affect the vascular compartment by secreting anti-angiogenic and vasoconstrictive mediators, including thrombospondin-1 and endothelin-1, leading to microvascular rarefaction and impaired perfusion [38,39]. In metabolic and diabetic cardiomyopathies, fibroblast subsets enriched for glycolytic flux and altered mitochondrial oxidative metabolism link metabolic adaptation to sustained fibrotic activity, underscoring the intersection between energetic adaptation and matrix persistence [23,29,40].

Beyond these intrinsic alterations with myocytes, fibroblasts can perpetuate disease through reciprocal signaling with immune cells and endothelial cells. Chronic fibroblast states engage in sustained ligand-receptor interactions with macrophages and endothelium through signaling pairs such as CXCL12-atypical chemokine receptor 3 (ACKR3) and midkine (MDK)-LDL-related protein 1 (LRP1), maintaining low-level inflammation and continuous ECM turnover [41,42,43,44]. Single-nucleus RNA-seq of failing human myocardium has identified fibroblast clusters expressing an immunomodulatory phenotype that perpetuates cytokine signaling and leukocyte recruitment [42]. These paracrine networks blur the boundary between inflammatory and fibrotic phases, converting fibroblasts from transient repair mediators into central components of the chronic remodeling environment.

### 2.4. Heterogeneous Fibroblast Populations in Heart Diseases

Cross-organ single-cell and spatial atlases in human show that fibroblast identity is highly conserved across tissues and shaped by local context [45,46]. Populations analogous to adventitial *PI16*^+^ and interstitial *COL15A1*^+^ fibroblasts are observed in heart, lung, kidney, and gut in human cross-organ atlases, with activation signatures conserved across mouse and human injury models, namely fibroblast activation characterized by upregulation of *ACTA2*, *POSTN*, *CTHRC1*, and collagen genes [45,47,48]. These findings indicate that many CF markers reflect a broader stromal activation pattern rather than a uniquely cardiac phenotype (Table 1). For cardiac disease, this reframes how marker expression is interpreted, suggesting fibroblast activation, but not necessarily cardiac specificity or pathogenic potential.

Within the myocardium, conserved activation patterns are modified by mechanical load, electrophysiologic signaling, and spatial niche, producing context-dependent fibroblast states [22]. After MI or pressure overload, activated fibroblasts accumulate in peri-infarct regions where TGF-β/SMAD and YAP/TAZ signaling predominate [26,27]. These cells co-express canonical fibrotic markers with heart-relevant paracrine factors such as IL-33, CXCL12, and insulin-like growth factor 1 (IGF1) that influence CM survival, angiogenesis, and immune recruitment [43,49,50]. Perivascular-derived fibroblast stromal cells and subepicardial fibroblasts further diversify this landscape post MI: the former aligns with angiogenic support functions, whereas the latter retains developmental regulators such as *WT1* and *TBX18*, consistent with their lineage history and proximity to the epicardial surface [35,42,51].

Marker overlap across organs complicates disease interpretation. For example, *POSTN* is transiently induced in CFs during scar formation but remains chronically elevated in pulmonary and renal fibrosis [52,53]. *CTHRC1* marks late-stage fibroblasts in the myocardium, where it contributes to scar stabilization, but is associated with invasive or angiogenic phenotypes in other tissues [48,54]. Even structural collagen genes are subject to distinct regulatory control—CFs rely primarily on TGF-β/SMAD3-responsive elements within *COL1A1* [7,32], whereas lung fibroblasts depend on interleukin-11 (IL-11)/signal transducer and activator of transcription-3 (STAT3)-driven enhancers [55,56]. *ACTA2* exemplifies the temporal restriction of fibroblast activation, following MI or pressure overload, α-SMA^+^ fibroblasts transiently mediate wound contraction before maturing into *CTHRC1*^+^/*LOX*^+^ matrifibrocytes [9,22,25,54]. In pulmonary and vascular fibrosis, however, *ACTA2* expression persists, promoting chronic stiffness [54]. Single-cell assay for transposase-accessible chromatin using sequencing (ATAC-seq) analyses indicate dynamic accessibility changes at profibrotic loci in cardiac non-myocytes after MI, whereas fibroblasts in systemic sclerosis exhibit broad, disease-associated chromatin remodeling at non-fibrotic or regulatory loci, consistent with a shift toward a pathogenic state [23,47,57].

Additional examples highlight how fibroblast-shared markers acquire distinct, tissue-specific functions. Fibroblast activation protein (FAP) marks activated CFs that emerge after injury and represent a therapeutically targetable population [58]. Activated fibroblasts engage paracrine pathways involving IL-33, CXCL12, and IGF-1 that support CM survival and angiogenesis [43,49,50]. In tumors or hepatic fibrosis, by contrast, FAP^+^ fibroblasts are immunosuppressive and matrix-degrading [59]. *TCF21*, a cardiac-lineage determinant, reemerges in reparative *TCF21*^+^/*CXCL12*^+^ fibroblasts after injury but is largely absent from adult fibroblasts in other organs, highlighting its relative cardiac enrichment [9,13]. Furthermore, *GLI1*^+^ perivascular stromal cells expand after injury and differentiate into *ACTA2*^+^*, POSTN*^+^, and *COL1A1*^+^ myofibroblasts in the heart, occupying an intermediate transcriptional state expressing platelet-derived growth factor receptor beta (PDGFRβ) and ECM-remodeling genes such as *CTHRC1* and *LOX* [9,20,27]. Their activation is modulated by Hedgehog signaling and mechanical stress, contributing to scar stabilization acutely but promoting chronic fibrosis if persistent [60]. This duality parallels the transient versus sustained activation patterns observed for *POSTN* and *ACTA2*, emphasizing that context dictates whether fibroblast activation is reparative or pathogenic.

Disease context further modulates fibroblast composition. Ischemic cardiomyopathy features expansion of *POSTN*^+^/*CTHRC1*^+^/*LOX*^+^ matrifibrocytes in border zones, linked to collagen crosslinking and conduction heterogeneity [9,21,22]. Pressure overload and hypertensive HF favor proliferative *MYC*^+^/*CTGF*^+^/integrin β1 (*ITGB1*)^+^ fibroblasts with enhanced mechano-transduction [18,26,27]. Fibroblasts in HF with preserved ejection fraction (HFpEF) display mixed inflammatory-fibrotic phenotypes enriched for *ANGPTL4*, *SERPINE1*, and *TIMP3*, resembling activation patterns seen in diabetic nephropathy and pulmonary hypertension [54,61,62,63,64,65]. Metabolic cardiomyopathy, particularly in diabetes, generates fibroblasts characterized by oxidative-phosphorylation and glutathione-metabolism signatures, linking mitochondrial adaptation to sustained fibrosis [40,65].

From a translational standpoint, recognizing this cross-organ conservation has important implications. Cardiac-selective targeting will likely require combinatorial definitions that integrate lineage identifiers (e.g., *TCF21*, PDGFRα) with spatial localization and enhancer-based regulatory features [13,23,32,66]. Such multidimensional frameworks can distinguish transient reparative fibroblast states from persistent fibrogenic ones and enable interventions that modulate pathogenic CFs without disrupting essential stromal functions in other organs.

## 3. Current Therapeutic Strategies for Reducing or Reversing Cardiac Fibrosis

Advances from single-cell and spatial analyses have transformed our understanding of cardiac fibrosis from a uniform process to one driven by diverse fibroblast populations with distinct temporal and spatial roles. This recognition has reshaped therapeutic goals from broadly suppressing fibroblast activation to precisely modulating the subsets that sustain chronic remodeling while preserving those essential for repair. Current experimental and preclinical efforts, therefore, aim either to reduce fibrosis by attenuating maladaptive fibroblast activity or to reverse fibrosis by reprogramming or replacing fibrotic tissue with functional myocardium.

**Figure 2:**
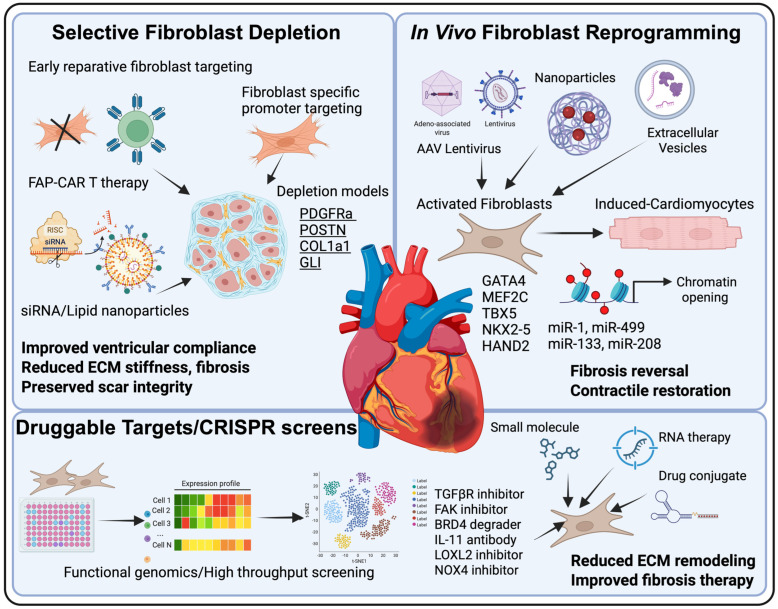
Therapeutic strategies targeting cardiac fibroblast plasticity. Schematic overview of approaches to attenuate or reverse cardiac fibrosis. Selective fibroblast depletion (FAP–chimeric antigen receptor T [CAR T], small interfering RNA (siRNA)/lipid nanoparticles (LNP), promoter-based ablation) reduces ECM stiffness while preserving scar integrity. In vivo reprogramming (*GATA4*, *MEF2C*, *TBX5*, *NKX2-5*, *HAND2*; miR-1, miR-133, miR-208, miR-499) converts activated fibroblasts into CMs, promoting fibrosis reversal and contractile restoration. Functional genomics and clustered regularly interspaced short palindromic repeats (CRISPR)-based screens identify druggable targets—TGF-βR, focal adhesion kinase (FAK), bromodomain-containing protein 4 (BRD4), *LOXL2*, NADPH oxidase 4 (*NOX4*) for precision antifibrotic therapy.

Approaches to achieve these outcomes can be broadly grouped into three categories (Figure 2): (1) specific depletion of pathogenic fibroblast subtypes that sustain chronic scarring; (2) in vivo reprogramming strategies that directly convert resident fibroblasts into CM-like cells to restore contractile function, and (3) identification of potential druggable targets and functional screening platforms to discover pharmacologic or genetic modulators of fibroblast plasticity. Each of these interventions carries distinct advantages and limitations related to selectivity, reversibility, and off-target effects. The following subsections examine these approaches in detail, beginning with methods that aim for selective fibroblast depletion, an approach that seeks to remove the most pathogenic fibroblast populations while sparing reparative or homeostatic stromal cells.

### 3.1. Specific Depletion of Pathological Fibroblast Subtypes

Fibroblast ablation has emerged as a promising therapeutic strategy to limit pathological remodeling and is now being refined through insights from single-cell and spatial atlases that define distinct fibroblast subtypes and states. Early studies in murine models used broadly expressed fibroblast promoters such as *COL1A2*-CreERT, *POSTN*-CreERT, or *PDGFRα*-CreERT established the principle that fibroblast removal must be both temporal and subtype-specific [67,68,69,70]. Complete depletion of fibroblasts during or immediately after MI caused ventricular rupture, demonstrating that reparative fibroblasts are necessary for scar stabilization [67]. In contrast, delayed or partial ablation reduced collagen crosslinking and limited adverse remodeling, emphasizing that fibroblast depletion is beneficial only after mechanical integrity has been reestablished [68,69].

Mouse models employing diphtheria-toxin receptor (DTR) ablation or suicide-gene systems have provided mechanistic insight into this timing and lineage dependence [67,68,69]. For example, *POSTN*-DTR mice enable the transient elimination of *POSTN*-expressing fibroblasts during the proliferative phase after injury [68]. Ablation in this window reduces scar stiffness and collagen density, but excessive depletion promotes dilation [68]. Broader ablation using *COL1A1*-diphtheria toxin A (DTA) or *PDGFRα*-DTA lines effectively decreases ECM content yet frequently compromises ventricular structure [69]. However, more selective approaches, such as *CTHRC1*-CreERT2 mice targeting late-stage matrifibrocytes, demonstrate reduced tissue stiffness and collagen deposition but with an increased mortality rate due to cardiac rupture [30]. These studies collectively show that depletion efficacy depends on timing and lineage restriction rather than absolute cell loss.

Building on these genetic proofs of concept, several molecular and immunologic strategies seek to achieve fibroblast removal through cell-surface or microenvironmental cues [58,71]. FAP, a membrane serine protease upregulated in activated fibroblasts across multiple organs, has become a prototypic target [6]. Antibody-drug conjugates and CAR T cells directed against FAP have achieved potent fibroblast clearance in preclinical cardiac and pulmonary fibrosis models [72,73]. In mice with pressure overload-induced cardiac fibrosis, adoptive transfer of FAP-CAR T cells decreased collagen deposition and improved ventricular compliance [58]. Yet systemic depletion of FAP^+^ cells can impair normal wound healing and hematopoietic niches, underscoring the need for spatially restricted or temporary activation systems such as drug-inducible CARs or localized nanoparticle delivery [74,75,76].

Similarly, genetic ablation or pathway inhibition of perivascular mesenchymal *GLI1*^+^ derived myofibroblasts have shown region-specific fibrosis attenuation during the subacute, pro-proliferative window [20]. Other potential targets include *THY1* (CD90), and PDGFRα, which are enriched in fibrogenic CF subsets [66,77]. Ligand–toxin conjugates and bispecific antibodies directed at these receptors have produced fibroblast-selective cytotoxicity in vitro, while LNP delivery of mRNAs or siRNA regulating fibrotic pathways without permanent ablation, offering a controllable middle ground between depletion and modulation [78,79].

The central limitation of depletion approaches lies in distinguishing pathological fibroblasts from those necessary for repair. Pathogenic and reparative subsets share many ECM and stress-response genes, leaving few unique molecular handles [80]. The optimal therapeutic window is narrow: too early and scar stability is compromised, too late and removal has minimal impact once collagen is crosslinked. During the early post-MI period, activated fibroblasts are essential for stabilizing the infarcted wall through provisional ECM deposition; premature depletion can compromise scar integrity, increasing the risk of wall thinning and ventricular rupture [22,30,67]. In contrast, persistent fibroblast activation in the chronic phase drives excessive matrix accumulation and ventricular stiffening, highlighting the narrow therapeutic window for safe intervention [6]. Off-target toxicity also remains a concern, as markers such as FAP and PDGFRα are expressed in other stromal or progenitor populations [81,82]. Moreover, many fibroblast markers are shared across stromal populations in other organs, including the lungs, liver, and kidneys [46]. Therefore, systemic depletion studies remain in the pre-clinical stages. These strategies raise concerns regarding unintended disruption of homeostatic fibroblast populations that are essential for tissue maintenance, immune regulation, and wound healing in non-cardiac organs. Such off-target effects could manifest as impaired organ repair, fibrosis dysregulation, or chronic inflammation outside the heart. Long-term safety remains another critical concern for fibroblast depletion strategies, as irreversible loss of fibroblast populations may impair adaptive remodeling, underscoring the need for transient, tightly regulated, and spatially controlled interventions. Continued integration of single-cell atlases, enhancer-level regulatory maps, and targeted delivery technologies will be critical to define fibroblast subsets that can be safely removed without compromising cardiac structure or systemic connective tissue homeostasis.

### 3.2. In Vivo Reprogramming: Rewiring Fibrosis Toward Functional Cardiac Regeneration

In vivo cardiac reprogramming seeks to regenerate contractile myocardium by converting resident CFs directly into induced cardiomyocyte (iCM)-like cells within the injured heart [83]. Unlike in vitro or transplantation-based approaches, this strategy operates in the native microenvironment, utilizing the spatial abundance and reparative localization of fibroblasts in the infarct and border zones [84]. By inducing lineage conversion in situ, in vivo reprogramming aims to bypass major limitations of cell therapy, including poor engraftment, arrhythmogenic risk, and immune rejection [85].

Lineage-tracing studies have confirmed that subsets of CFs can undergo reprogramming following enforced expression of cardiogenic transcription factor combinations such as *GATA4-MEF2C-TBX5* (GMT) or *GATA4-HAND2-MEF2C-TBX5* (GHMT) [86]. Conversion of these fibroblasts into iCMs improves structural integrity and partially restores ventricular function in experimental models of MI [86,87]. However, reprogramming outcomes are strongly influenced by the cellular origin and activation state of the fibroblasts [10,88]. Quiescent or early-activated fibroblasts exhibit greater responsiveness than myofibroblasts, whose cytoskeletal tension and compact chromatin limit transcription factor accessibility [89,90]. Age is also a major determinant: aged fibroblasts show reduced reprogramming efficiency due to oxidative stress, diminished *GATA4* expression, and epigenetic silencing of cardiogenic enhancers [91,92]. Strategies that restore youthful metabolic states or enhance autophagy have been shown to improve conversion efficiency, suggesting that rejuvenation of fibroblast physiology may expand the therapeutic window for reprogramming [84].

At the molecular level, transcription factors, miRNAs, and chromatin regulators cooperate to reestablish cardiac gene networks in vivo [93,94]. The GMT or GHMT combinations activate enhancer landscapes that mirror embryonic cardiogenesis, while cardiac miRNAs promote sarcomeric maturation and electrophysiologic competence [24,94,95,96,97]. Additional factors have been identified that enhance chromatin accessibility and stabilize cardiogenic transcriptional circuits [98]. These findings demonstrate that effective cardiac reprogramming requires both transcriptional activation and concurrent remodeling of the chromatin landscape.

The tissue microenvironment exerts equally significant control over reprogramming outcomes. Angiogenic factors such as fibroblast growth factor 2 (FGF2) and VEGFA facilitate conversion by improving perfusion and supporting metabolic remodeling, whereas inflammatory mediators, particularly interferon-β derived from macrophages, suppress reprogramming through paracrine inhibitory loops [99,100]. Co-administration of pro-angiogenic or immune-modulatory agents can therefore augment conversion efficiency and promote integration of newly generated iCMs. Single-cell transcriptomic analyses reveal that only a subset of reprogrammed fibroblasts achieve complete CM identity, while many cells stall in partially reprogrammed states that nonetheless contribute to improved ventricular function [101].

Delivery systems have emerged as a major determinant of translational success. Early studies employed retroviral and lentiviral vectors, but contemporary approaches favor adeno-associated virus (AAV) systems for their cardiac tropism and non-integrating characteristics [102]. Fibroblast-specific promoters such as *POSTN* have been incorporated into AAV constructs to restrict transgene expression to activated CFs and reduce off-target effects [103]. Engineered AAV9 and AAV-DJ vectors carrying GHMT or modified transcription factors with myocardin transactivation domains have improved conversion efficiency and enhanced left ventricular ejection fraction in murine infarction models [83,103]. Non-viral systems, including cationic gold nanoparticles, PLGA-PEI nanocarriers, and mesoporous silica nanoparticles functionalized with fibroblast-targeting peptides, have shown comparable success in delivery of plasmid or miRNA cargo to infarcted myocardium, reducing fibrosis and improving contractile function [72,83,102]. More recently, extracellular vesicle (EV)-based delivery derived from embryonic cardiogenic cells has been used to transport miRNAs to CFs, inducing partial reprogramming and promoting neovascularization [72,83,102]. These delivery systems provide a scalable, potentially less immunogenic alternative to viral vectors.

Collectively, these findings highlight that in vivo reprogramming is a multifactorial process driven by fibroblast state, transcriptional context, and delivery precision. Future refinements are expected to incorporate AI-derived design and small-molecular screening to identify human-specific reprogramming drivers, and AAV capsid engineering for enhanced CF tropism. As these tools progress, in vivo fibroblast reprogramming represents a promising therapeutic strategy to restore myocardial function by rebuilding tissue directly within the injured heart.

### 3.3. Potential Druggable Targets for Cardiac Fibrosis Therapy

Single-cell and multi-omic characterization of CFs has enabled systematic identification of molecular nodes that sustain fibrotic remodeling [104]. Earlier antifibrotic strategies focused on broad inhibition of TGF-β or renin-angiotensin signaling, whereas recent discovery platforms now combine phenotypic screening, CRISPR, functional genomics, and human induced pluripotent stem cell (iPSC)-based disease models to uncover context-specific regulators of fibroblast activation, metabolism, and matrix turnover [105].

High-content small-molecule screens using human iPSC-derived CFs and engineered cardiac tissues have identified several pharmacologically tractable pathways [105,106]. Automated phenotyping and transcriptomic profiling highlight inhibitors of TGF-β/SMAD3, YAP/TAZ, and endoplasmic reticulum (ER)-stress-IL-11 signaling as potent suppressors of fibroblast contractility and collagen synthesis [105]. Metabolic screening has revealed that inhibition of glutaminase (*GLS1*) or *NOX4* decreases profibrotic gene expression while maintaining fibroblast viability, defining metabolic checkpoints that modulate matrix deposition [107,108]. Patient-specific screening systems integrating multi-omic readouts further identified inflammatory co-receptors, such as myeloid differentiation factor 2 (MD2)/toll-like receptor 4 (TLR4), as drivers of fibroblast activation with pharmacologic blockade reducing collagen accumulation in engineered heart tissues and murine infarction models [106]. Recent computational biology studies on fibroblast-related gene regulatory networks have found genes such as integrin alpha L (ITGAL) and dual specificity phosphatase (DUSP) associated with the TGF-β/SMAD pathway as potential druggable targets to blunt human cardiac fibrosis [109].

Transcriptomics and phospho-proteomic profiling studies have revealed several druggable nodes such as FAK-YAP/TAZ mechanotransduction [27], IL-11–STAT3 signaling [110], and LOXL2-mediated collagen crosslinking [111]. Several of these pathways already have inhibitors in clinical development for non-cardiac fibrotic disorders, including BRD4 degraders, FAK inhibitors, and IL-11 neutralizing antibodies, offering opportunities for repurposing in cardiac disease [112,113,114,115]. Overall, the convergence of single-cell atlases, CRISPR functional screens, and high-throughput drug discovery defines a framework for precision antifibrotic therapy. By aligning chemical, genetic, and RNA-based disturbances with state-resolved fibroblast biology, these approaches are transforming the treatment of cardiac fibrosis from broad pathway inhibition to cell-state-specific modulation grounded in mechanistic and translational precision.

## 4. Limitations and Translational Challenges

While fibroblast-based antifibrotic and regenerative therapies hold significant promise for cardiac repair, several scientific, technical, and translational barriers must be addressed before clinical implementation is feasible. Despite considerable progress in direct cardiac reprogramming, it still exhibits relatively low efficiency in adult hearts. Fibroblast-specific epigenetic marks, including DNA methylation and H3K27 trimethylation, limit chromatin accessibility and suppress CM lineage gene expression [113,116]. The persistence of profibrotic signaling pathways, including TGF-β/SMAD and RhoA-ROCK cascades, further reinforces chromatin states that are resistant to reprogramming [26,32,117]. In addition to epigenetic barriers, direct cardiac reprogramming is actively suppressed by inflammatory signaling within the injured myocardium. Pro-inflammatory cytokines and chemokines, including IL-1α, IL-2, IL-26, and CXCL family members, as well as the inflammatory transcription factor C/EBPβ, reinforce fibroblast identity and impede activation of CM gene programs, making immune activation a significant barrier to reprogramming in adult hearts [117]. Emerging strategies that combine epigenetic modulators, chromatin remodeling approaches, optimized reprogramming cocktails, and targeted immune-suppressive or anti-inflammatory interventions may help overcome these transcriptional and structural barriers.

Another major risk associated with in vivo reprogramming is the potential generation of electrically immature iCM-like cells [118]. These CM-like cells exhibit immature electrophysiological properties, incomplete sarcomere organization, and irregular calcium handling, which may increase arrhythmogenic risk when introduced into diseased myocardium [118]. Heterogeneous or incomplete conversion may exacerbate conduction abnormalities, particularly within infarct border zones. Similarly, fibroblast-depletion strategies require precise temporal control, as premature elimination of reparative fibroblasts during the acute injury phase risks ventricular rupture, whereas delayed intervention may provide limited benefit once fibrosis has stabilized.

Ensuring safety and minimizing immune activation remain key challenges in the translation of fibroblast-targeted gene and RNA therapies [84]. Viral vectors achieve high transduction efficiency but are constrained by immunogenicity, limited payload capacity, and challenges in restricting expression to fibroblast subpopulations. Viral delivery systems, particularly lentivirus and retrovirus, enable efficient gene transfer but pose risks of insertional mutagenesis, long-term vector persistence, and dose-dependent hepatotoxicity [84,102,103,116]. Although largely episomal, AAV vectors can still provoke cytotoxic immune responses [84,85,103]. Moreover, achieving spatially uniform and fibroblast-specific delivery is hindered by infarct heterogeneity, dense ECM, and shared marker expression across stromal populations in multiple organs, increasing the risk of off-target effects [116].

Furthermore, species-specific differences between murine and human myocardium present a major translational barrier [84,85,102]. Mouse models differ substantially in cardiac size, mechanical load, fibroblast composition, immune responses, and regenerative capacity, potentially overestimating therapeutic efficacy and underestimating safety risks. The therapeutic efficacy and safety observed in mouse models may not reliably predict human outcomes. In particular, strategies involving fibroblast depletion or in vivo reprogramming raise distinct concerns in the human heart, where altered conduction properties, limited regenerative plasticity, and heterogeneous scar architecture may amplify arrhythmogenic or structural risks. Large-animal studies and human tissue-based validation will therefore be essential to evaluate vector distribution, immune tolerance, electrophysiologic integration, and long-term safety under clinically relevant conditions.

Finally, the long-term safety of transcriptional and RNA-editing strategies, including CRISPR-based gene modulation and ADAR-mediated RNA editing, requires rigorous evaluation. Off-target activity could disrupt endogenous gene networks, emphasizing the need for transient, tightly regulated expression systems and comprehensive off-target profiling using high-depth sequencing approaches. Collectively, these considerations indicate that clinical feasibility depends not only on regenerative potential but also on precise temporal control, immune tolerance, and electrophysiologic safety.

## 5. Future Directions: Convergences of New Drug Discovery and Gene Delivery Approaches

Despite major progress in defining CF heterogeneity and activation states, therapeutic translation remains limited by the inability to selectively modulate pathogenic fibroblast subsets without disrupting normal repair. Future strategies will likely arise from the combination of precision drug discovery and targeted gene delivery, integrating advances in molecular profiling, medicinal chemistry, and vector engineering.

Next-generation small-molecule and biologic therapies are shifting from broad antifibrotic suppression toward pathway-specific modulation. Recent studies have begun to identify inhibitors that selectively disrupt signaling nodes such as TGF-β receptor I kinase [119,120,121], YAP/TAZ-transcriptional enhancer associate domain (TEAD) interaction [122], and LOX-mediated matrix crosslinking [123]. However, their efficacy in reducing cardiac fibrosis needs further scientific exploration. Epigenetic modulators are emerging as complementary tools to overcome stable profibrotic chromatin states. Modulation of sirtuin signaling and other chromatin-associated regulators has been shown to suppress fibroblast activation by integrating metabolic rewiring with transcriptional control, suggesting a strategy to indirectly destabilize fibrotic gene programs while preserving cellular viability [113]. Further exploration of targeted modulation of noncoding RNAs that regulate the fibroblast-to-myofibroblast transition, including lncRNAs such as *H19X* and *GAS5* and circRNAs such as *circHIPK3*, may enable selective destabilization of profibrotic fibroblast states without impairing essential reparative responses [124]. Combining these pharmacologic agents with context-dependent biomarkers could enable titratable, reversible antifibrotic control tailored to patient-specific fibroblast phenotypes.

Distinct from antifibrotic approaches, emerging reprogramming strategies leverage epigenetic remodeling and suppression of inflammatory signaling to improve direct cardiac reprogramming efficiency. Histone readers such as PHF7 cooperate with cardiac super-enhancer-associated SWI/SNF chromatin remodeling complexes to enhance cardiogenic transcriptional accessibility [98]. Pharmacologic inhibition of the polycomb group protein BMI1 using PTC-209 further improves reprogramming efficiency in adult cardiac fibroblasts by downregulating immune–inflammatory pathways, including JAK/STAT3 and MAPK/ERK1/2 signaling [117]. Notably, combinatorial epigenetic and anti-inflammatory strategies, such as the IMAP cocktail (IGF-1, MM589, A83-01, and PTC-209), further enhance GMT-mediated reprogramming by suppressing C–C chemokine signaling, resulting in robust induction of cardiac gene programs and repression of fibroblast identity [117].

Additionally, emerging gene- and RNA-based delivery systems offer unprecedented control over fibroblast behavior [125]. Advances in AAV engineering, LNPs, and hybrid polymer carriers can potentially be combined with cell-type-restricted transgene expression using promoters and enhancer elements active primarily in CF subpopulations. These platforms can deliver siRNA, mRNA, or CRISPR effectors to silence profibrotic mediators to reprogram fibroblasts toward reparative or contractile fates. This integration of single-cell-defined enhancer maps with synthetic promoter design may further enable context-dependent gene delivery, ensuring that therapeutic modulation occurs only within specific fibroblast states and at precise stages of injury and repair.

Ultimately, the convergence of rational drug discovery and precision gene delivery is expected to transform cardiac fibrosis therapy from nonspecific inhibition to programmable modulation of fibroblast function. Integrating these approaches within regenerative frameworks, where antifibrotic agents stabilize the matrix environment and gene-based tools restore cellular function, will be essential to achieve durable myocardial repair and true reversal of fibrotic disease.

## 6. Conclusions

Cardiac fibrosis remains a defining component in HF, where the cellular mechanisms that enable repair ultimately drive progressive remodeling and loss of function. Insights from single-cell and spatial analyses have revealed that fibroblast activation is not a uniform reaction, but a temporally and spatially regulated process with therapeutic entry points at multiple stages of disease. Emerging evidence of fibroblast plasticity, the capacity to reversibly transition between quiescent, activated, and reparative states, provides a fundamental rationale for designing therapeutics aimed at reprogramming or functionally redirecting these cells rather than merely depleting them. Translating these discoveries into treatment will require integrating antifibrotic strategies that interrupt chronic signaling and matrix accumulation with regenerative approaches that restore myocardial function through fibroblast reprogramming or paracrine modulation. Progress will depend on precise delivery systems, state-specific biomarkers, and disease-stage modeling that allow selective targeting of pathogenic fibroblast subsets without impairing normal repair. As these experimental and computational frameworks mature, they have the potential to redefine cardiac fibrosis as a reversible process, guiding the development of therapies that not only prevent disease progression but also promote functional recovery in HF.

## Figures and Tables

**Table 1:** Summary of fibroblast populations or states.

Subpopulation	Developmental Origin/Disease State	Key Markers	Functional Role
Quiescent resident fibroblasts	Epicardial-derived mesenchyme (primarily) minor endocardial contribution	PDGFRα, *TCF21*, *VIM*, CD90, SCA-1, *DCN*, *LUM*	Maintain ECM homeostasis, mechanical support, and tissue integrity
Early-activated fibroblasts/reparative fibroblasts	Acute injury or inflammation-induced activation of resident fibroblasts	*TCF21*^+^, *POSTN*^+^ (transient), IL6, VEGFA, ANGPT1	Initiate reparative activation, promote angiogenesis, immune signaling, and provisional ECM synthesis
Reparative fibroblasts/paracrine modulators	Injury-responsive	*COL1A1*, *COL3A1*, *FN1* (ED-A fibronectin), IGF-1, HGF, VEGF, FGF2, microRNA (miRNA) cargo (e.g., miR-21, miR-29)	Promote angiogenesis, CM survival, and resolution of inflammation
Activated myofibroblasts	Injury-induced differentiation of resident fibroblasts	α-SMA (*ACTA2*), *POSTN*, *COL1A1*, *COL3A1*, *FN1*	Drive scar formation, ECM deposition, and wound contraction
Pro-fibrotic fibroblasts (persistent activation) matrifibroblast	Chronic injury, aging myocardium	*CTHRC1*, *LOX*, *TIMP1*, *DDR2*, CTGF, TGF-β	Promote pathological fibrosis, collagen crosslinking, and ventricular stiffening
Epicardial-derived fibroblasts	Embryonic epicardium	*WT1*, *TBX18*, *TCF21*, PDGFRα	Contribute to developmental fibroblast lineages’ contribution, reactivated post-injury
Perivascular fibroblasts	Pericyte-derived or adventitial mesenchyme	PDGFRβ, NG2, CD146, *GLI1*, *RGS5*	Regulate microvascular remodeling and endothelial-stromal interactions
Inflammatory/ immune-interacting fibroblasts	Chronic HF, autoimmune disease, or systemic fibrosis	CCL19, CCL21, CXCL10, IL-33, CXCL12, MHC-II, MDK, LRP1	Sustain chronic inflammation via macrophage and endothelial crosstalk, reinforce ECM turnover

## Data Availability

No new data was created in this review article.

## Abbreviations

The following abbreviations are used in this manuscript:

AAV	Adeno-associated virus
AAV9/AAV-DJ	Adeno-associated virus serotype 9/DJ capsid variant
ACKR3	Atypical chemokine receptor 3
ACTA2 (α-SMA)	Alpha smooth muscle actin
ACE/ACEi	Angiotensin-converting enzyme/ACE inhibitor
ANGPT1	Angiopoietin-1
ANGPTL4	Angiopoietin-like 4
ATAC-seq	Assay for Transposase-Accessible Chromatin using sequencing
β-blocker	Beta adrenergic receptor blocker
BRD4	Bromodomain-containing protein 4
CAR	Chimeric antigen receptor
CCL19/CCL21	C-C motif chemokine ligand 19/21
CD146 (MCAM)	Melanoma cell adhesion molecule
iCMs	Induced cardiomyocyte-like cells
CF(s)	Cardiac fibroblast(s)
CMs	Cardiomyocytes
COL1A1/COL1A2/COL3A1	Collagen type I alpha-1/alpha-2, type III alpha-1
COL15A1	Collagen type XV alpha-1
CRISPR	Clustered Regularly Interspaced Short Palindromic Repeats
CTGF	Connective tissue growth factor
CTHRC1	Collagen triple helix repeat-containing 1
CTCF	CCCTC-binding factor
CXCL10/CXCL12	C-X-C motif chemokine ligand 10/12
DCN	Decorin
dCas9	Dead Cas9
DDR2	Discoidin domain receptor 2
DNA	Deoxyribonucleic acid
DTA	Diphtheria toxin A
DTR	Diphtheria toxin receptor
DUSP	Dual specificity phosphatase (family)
ECM	Extracellular matrix
ED-A fibronectin (FN1-ED-A)	Extra-domain A fibronectin
EMT	Epithelial-to-mesenchymal transition
EndoMT	Endothelial-to-mesenchymal transition
EP300 (p300), KAT5 (TIP60), SRCAP	Chromatin regulators
ER	Endoplasmic reticulum
EV(s)	Extracellular vesicle(s)
FAK	Focal adhesion kinase
FAP	Fibroblast activation protein-α
FGF2	Fibroblast growth factor 2
FSP1 (S100A4)	Fibroblast-specific protein 1
GATA4	GATA-binding protein 4
GHMT	GATA4–HAND2–MEF2C–TBX5 (reprogramming cocktail)
GMT/GMT-H	GATA4, MEF2C, TBX5 (and HAND2)
GLI1	GLI family zinc finger 1
GLS1	Glutaminase 1
HAND2	Heart And Neural Crest Derivatives Expressed 2
H3K27me3	Histone H3 lysine 27 trimethylation
HGF	Hepatocyte growth factor
HF	Heart failure
HFpEF	Heart failure with preserved ejection fraction
iPSC(s)	Induced pluripotent stem cell(s)
IGF1	Insulin-like growth factor 1
IL-1β/IL-6/IL-11	Interleukin-1 beta/Interleukin-6/Interleukin-11
IL-33	Interleukin-33
ITGAL	Integrin alpha L (CD11a)
ITGB1 (ITGβ1)	Integrin beta-1
LNP(s)	Lipid nanoparticle(s)
LOX	Lysyl oxidase
LOXL2	Lysyl oxidase-like 2
lncRNA	Long non-coding RNA
LRP1	LDL receptor-related protein 1
LUM	Lumican
MD2	Myeloid differentiation factor 2
MDK	Midkine
MEF2C	Myocyte enhancer factor 2C
MI	Myocardial infarction
miRNA	MicroRNA
mRNA	Messenger RNA
MHC-II	Major histocompatibility complex class II
MYC	Myc proto-oncogene
NG2 (CSPG4)	Neuron-glial antigen 2/Chondroitin sulfate proteoglycan 4
NKX2-5	NK2 homeobox 5
NOX4	NADPH oxidase 4
PDGFRα	Platelet-derived growth factor receptor alpha
PDGFRβ	Platelet-derived growth factor receptor beta
PI16	Peptidase inhibitor 16
PLGA-PEI	Poly(lactic-co-glycolic acid)–polyethyleneimine (nanocarrier)
POSTN	Periostin
RGS5	Regulator of G-protein signaling 5
RNA	Ribonucleic acid
SCA-1	Stem cell antigen-1
SERPINE1 (PAI-1)	Serpin family E member 1
SMAD2/3	Mothers against decapentaplegic homolog 2/3 (TGF-β effectors)
STAT3	Signal transducer and activator of transcription 3
TBX18/TBX20/TBX5	T-box transcription factors 18/20/5
TEAD	Transcriptional enhanced associate domain
TCF21	Transcription factor 21
TGF-β	Transforming growth factor beta
TGF-βR1	TGF-β receptor type I
THY1 (CD90)	Thymocyte differentiation antigen 1
TIMPs/TIMP1/TIMP3	Tissue inhibitor(s) of metalloproteinases 1/3
TLR4	Toll-like receptor 4
TNF-α	Tumor necrosis factor alpha
VEGFA	Vascular endothelial growth factor A
VIM	Vimentin
WT1	Wilms tumor 1
WNT	Wnt signaling pathway
YAP/TAZ	Yes-associated protein/Transcriptional co-activator with PDZ-binding motif

## References

[1] Buja L.M. (2023). Pathobiology of Myocardial Ischemia and Reperfusion Injury: Models, Modes, Molecular Mechanisms, Modulation, and Clinical Applications. Cardiol. Rev..

[2] Haubner B.J., Schneider J., Schweigmann U., Schuetz T., Dichtl W., Velik-Salchner C., Stein J.-I., Penninger J.M. (2016). Functional Recovery of a Human Neonatal Heart After Severe Myocardial Infarction. Circ. Res..

[3] Moretti L., Stalfort J., Barker T.H., Abebayehu D. (2022). The interplay of fibroblasts, the extracellular matrix, and inflammation in scar formation. J. Biol. Chem..

[4] Rog-Zielinska E.A., Norris R.A., Kohl P., Markwald R. (2016). The Living Scar—Cardiac Fibroblasts and the Injured Heart. Trends Mol. Med..

[5] Moore-Morris T., Guimarães-Camboa N., Yutzey K.E., Pucéat M., Evans S.M. (2015). Cardiac fibroblasts: From development to heart failure. J. Mol. Med..

[6] Morfino P., Aimo A., Castiglione V., Gálvez-Montón C., Emdin M., Bayes-Genis A. (2023). Treatment of cardiac fibrosis: From neuro-hormonal inhibitors to CAR-T cell therapy. Heart Fail. Rev..

[7] Frangogiannis N. (2020). Transforming growth factor-β in tissue fibrosis. J. Exp. Med..

[8] Steele L., Olabi B., Roberts K., Mazin P.V., Koplev S., Tudor C., Rumney B., Admane C., Jiang T., Correa-Gallegos D. (2025). A single-cell and spatial genomics atlas of human skin fibroblasts reveals shared disease-related fibroblast subtypes across tissues. Nat. Immunol..

[9] Calcagno D.M., Taghdiri N., Ninh V.K., Mesfin J.M., Toomu A., Sehgal R., Lee J., Liang Y., Duran J.M., Adler E. (2022). Single-cell and spatial transcriptomics of the infarcted heart define the dynamic onset of the border zone in response to mechanical destabilization. Nat. Cardiovasc. Res..

[10] Chen Y., Yang Z., Zhao Z.-A., Shen Z. (2017). Direct reprogramming of fibroblasts into cardiomyocytes. Stem. Cell Res. Ther..

[11] Ali S.R., Ranjbarvaziri S., Talkhabi M., Zhao P., Subat A., Hojjat A., Kamran P., Müller A.M.S., Volz K.S., Tang Z. (2014). Developmental heterogeneity of cardiac fibroblasts does not predict pathological proliferation and activation. Circ. Res..

[12] Zhang J., Tao R., Campbell K.F., Carvalho J.L., Ruiz E.C., Kim G.C., Schmuck E.G., Raval A.N., da Rocha A.M., Herron T.J. (2019). Functional cardiac fibroblasts derived from human pluripotent stem cells via second heart field progenitors. Nat. Commun..

[13] Hu H., Lin S., Wang S., Chen X. (2020). The Role of Transcription Factor 21 in Epicardial Cell Differentiation and the Development of Coronary Heart Disease. Front. Cell Dev. Biol..

[14] Ivey M.J., Tallquist M.D. (2016). Defining the Cardiac Fibroblast. Circ. J..

[15] Zeisberg E.M., Tarnavski O., Zeisberg M., Dorfman A.L., McMullen J.R., Gustafsson E., Chandraker A., Yuan X., Pu W.T., Roberts A.B. (2007). Endothelial-to-mesenchymal transition contributes to cardiac fibrosis. Nat. Med..

[16] Snider P., Standley K.N., Wang J., Azhar M., Doetschman T., Conway S.J. (2009). Origin of Cardiac Fibroblasts and the Role of Periostin. Circ. Res..

[17] Harrington A., Moore-Morris T. (2024). Cardiac fibroblasts in heart failure and regeneration. Front. Cell Dev. Biol..

[18] Tsai C.-R., Martin J.F. (2022). Hippo signaling in cardiac fibroblasts during development, tissue repair, and fibrosis. Curr. Top. Dev. Biol..

[19] Chen M., Zhong J., Wang Z., Xu H., Chen H., Sun X., Lu Y., Chen L., Xie X., Zheng L. (2021). Fibroblast Growth Factor 21 Protects Against Atrial Remodeling via Reducing Oxidative Stress. Front. Cardiovasc. Med..

[20] Kramann R., Schneider R.K., DiRocco D.P., Machado F., Fleig S., Bondzie P.A., Henderson J.M., Ebert B.L., Humphreys B.D. (2015). Perivascular *Gli1*+ Progenitors Are Key Contributors to Injury-Induced Organ Fibrosis. Cell Stem. Cell.

[21] Nie W., Zhao Z., Xiahou Z., Zhang J., Liu Y., Wang Y., Wang Z. (2025). Single-cell RNA sequencing reveals the potential role of Postn(+) fibroblasts in promoting the progression of myocardial fibrosis after myocardial infarction. Sci. Rep..

[22] Forte E., Skelly D.A., Chen M., Daigle S., Morelli K.A., Hon O., Philip V.M., Costa M.W., Rosenthal N.A., Furtado M.B. (2020). Dynamic Interstitial Cell Response during Myocardial Infarction Predicts Resilience to Rupture in Genetically Diverse Mice. Cell Rep..

[23] Wang L., Yang Y., Ma H., Xie Y., Xu J., Near D., Wang H., Garbutt T., Li Y., Liu J. (2022). Single-cell dual-omics reveals the transcriptomic and epigenomic diversity of cardiac non-myocytes. Cardiovasc. Res..

[24] Hashimoto H., Wang Z., Garry G.A., Malladi V.S., Botten G.A., Ye W., Zhou H., Osterwalder M., Dickel D.E., Visel A. (2019). Cardiac Reprogramming Factors Synergistically Activate Genome-wide Cardiogenic Stage-Specific Enhancers. Cell Stem. Cell.

[25] Fu X., Khalil H., Kanisicak O., Boyer J.G., Vagnozzi R.J., Maliken B.D., Sargent M.A., Prasad V., Valiente-Alandi I., Blaxall B.C. (2018). Specialized fibroblast differentiated states underlie scar formation in the infarcted mouse heart. J. Clin. Investig..

[26] Khalil H., Kanisicak O., Prasad V., Correll R.N., Fu X., Schips T., Vagnozzi R.J., Liu R., Huynh T., Lee S.-J. (2017). Fibroblast-specific TGF-β-Smad2/3 signaling underlies cardiac fibrosis. J. Clin. Investig..

[27] Garoffolo G., Casaburo M., Amadeo F., Salvi M., Bernava G., Piacentini L., Chimenti I., Zaccagnini G., Milcovich G., Zuccolo E. (2022). Reduction of Cardiac Fibrosis by Interference with YAP-Dependent Transactivation. Circ. Res..

[28] Mitchell M.D., Laird R.E., Brown R.D., Long C.S. (2007). IL-1β stimulates rat cardiac fibroblast migration via MAP kinase pathways. Am. J. Physiol. Circ. Physiol..

[29] Wang F., Yin X., Fan Y.-M., Zhang X., Ma C., Jia K., Zhou W., Tang Z., Qi L.-W., Li J. (2023). Upregulation of glycolytic enzyme PFKFB3 by deubiquitinase OTUD4 promotes cardiac fibrosis post myocardial infarction. J. Mol. Med..

[30] Wang D., Zhang Y., Ye T., Zhang R., Zhang L., Shi D., Li T., Xia G., Niu K., Zhao Z. (2023). *Cthrc1* deficiency aggravates wound healing and promotes cardiac rupture after myocardial infarction via non-canonical WNT5A signaling pathway. Int. J. Biol. Sci..

[31] Zhang Q., Cai Z., Zhang Y. (2025). Understanding of the characteristics of fibroblasts in ischemic cardiomyopathy using single-nucleus RNA sequencing. Sci. Rep..

[32] Algeciras L., Palanca A., Maestro D., RuizdelRio J., Villar A.V. (2021). Epigenetic alterations of TGFβ and its main canonical signaling mediators in the context of cardiac fibrosis. J. Mol. Cell. Cardiol..

[33] Ge Z., Yin C., Li Y., Tian D., Xiang Y., Li Q., Tang Y., Zhang Y. (2022). Long noncoding RNA NEAT1 promotes cardiac fibrosis in heart failure through increased recruitment of EZH2 to the Smad7 promoter region. J. Transl. Med..

[34] Gill R., Lu D.R., Eres I., Lu J., Cui J., Wang C., Yu Z.J., Yamawaki T., Zhou H., Pei B. (2025). Dissecting regulatory non-coding GWAS loci reveals fibroblast causal genes with pathophysiological relevance to heart failure. Nat. Commun..

[35] Skelly D.A., Squiers G.T., McLellan M.A., Bolisetty M.T., Robson P., Rosenthal N.A., Pinto A.R. (2018). Single-Cell Transcriptional Profiling Reveals Cellular Diversity and Intercommunication in the Mouse Heart. Cell Rep..

[36] Yang J., Savvatis K., Kang J.S., Fan P., Zhong H., Schwartz K., Barry V., Mikels-Vigdal A., Karpinski S., Kornyeyev D. (2016). Targeting LOXL2 for cardiac interstitial fibrosis and heart failure treatment. Nat. Commun..

[37] Quinn T.A., Camelliti P., Rog-Zielinska E.A., Siedlecka U., Poggioli T., O’TOole E.T., Knöpfel T., Kohl P. (2016). Electrotonic coupling of excitable and nonexcitable cells in the heart revealed by optogenetics. Proc. Natl. Acad. Sci. USA.

[38] Frangogiannis N.G., Ren G., Dewald O., Zymek P., Haudek S., Koerting A., Winkelmann K., Michael L.H., Lawler J., Entman M.L. (2005). Critical Role of Endogenous Thrombospondin-1 in Preventing Expansion of Healing Myocardial Infarcts. Circulation.

[39] Shi-Wen X., Chen Y., Denton C.P., Eastwood M., Renzoni E.A., Bou-Gharios G., Pearson J.D., Dashwood M., du Bois R.M., Black C.M. (2004). Endothelin-1 Promotes Myofibroblast Induction through the ETA Receptor via a rac/Phosphoinositide 3-Kinase/Akt-dependent Pathway and Is Essential for the Enhanced Contractile Phenotype of Fibrotic Fibroblasts. Mol. Biol. Cell.

[40] Gibb A.A., Lazaropoulos M.P., Elrod J.W. (2020). Myofibroblasts and Fibrosis: Mitochondrial and Metabolic Control of Cellular Differentiation. Circ. Res..

[41] Chatterjee M. (2022). Atypical Roles of the Chemokine Receptor ACKR3/CXCR7 in Platelet Pathophysiology. Cells.

[42] Koenig A.L., Shchukina I., Amrute J., Andhey P.S., Zaitsev K., Lai L., Bajpai G., Bredemeyer A., Smith G., Jones C. (2022). Single-cell transcriptomics reveals cell-type-specific diversification in human heart failure. Nat. Cardiovasc. Res..

[43] Dã¶Ring Y., Pawig L., Weber C., Noels H. (2014). The CXCL12/CXCR4 chemokine ligand/receptor axis in cardiovascular disease. Front. Physiol..

[44] Hulsmans M., Clauss S., Xiao L., Aguirre A.D., King K.R., Hanley A., Hucker W.J., Wülfers E.M., Seemann G., Courties G. (2017). Macrophages Facilitate Electrical Conduction in the Heart. Cell.

[45] Gao Y., Li J., Cheng W., Diao T., Liu H., Bo Y., Liu C., Zhou W., Chen M., Zhang Y. (2024). Cross-tissue human fibroblast atlas reveals myofibroblast subtypes with distinct roles in immune modulation. Cancer Cell.

[46] Liu K., Cui Y., Han H., Guo E., Shi X., Xiong K., Zhang N., Zhai S., Sang S., Liu M. (2025). Fibroblast atlas: Shared and specific cell types across tissues. Sci. Adv..

[47] Lendahl U., Muhl L., Betsholtz C. (2022). Identification, discrimination and heterogeneity of fibroblasts. Nat. Commun..

[48] Ruiz-Villalba A., Romero J.P., Hernández S.C., Vilas-Zornoza A., Fortelny N., Castro-Labrador L., Martin-Uriz P.S., Lorenzo-Vivas E., García-Olloqui P., Palacios M. (2020). Single-Cell RNA Sequencing Analysis Reveals a Crucial Role for *CTHRC1* (Collagen Triple Helix Repeat Containing 1) Cardiac Fibroblasts After Myocardial Infarction. Circulation.

[49] Zhu J., Carver W. (2012). Effects of interleukin-33 on cardiac fibroblast gene expression and activity. Cytokine.

[50] Heinen A., Nederlof R., Panjwani P., Spychala A., Tschaidse T., Reffelt H., Boy J., Raupach A., Gödecke S., Petzsch P. (2019). IGF1 Treatment Improves Cardiac Remodeling after Infarction by Targeting Myeloid Cells. Mol. Ther..

[51] Fang M., Xiang F.-L., Braitsch C.M., Yutzey K.E. (2016). Epicardium-derived fibroblasts in heart development and disease. J. Mol. Cell. Cardiol..

[52] Naik P.K., Bozyk P.D., Bentley J.K., Popova A.P., Birch C.M., Wilke C.A., Fry C.D., White E.S., Sisson T.H., Tayob N. (2012). Periostin promotes fibrosis and predicts progression in patients with idiopathic pulmonary fibrosis. Am. J. Physiol. Lung Cell Mol. Physiol..

[53] Cho A., Jin W., Lee J., Shin N., Lee M.S., Li L., Yang S.H., Park K.S., Yang C.W., Kim D.K. (2023). Periostin deficiency attenuates kidney fibrosis in diabetic nephropathy by improving pancreatic β-cell dysfunction and reducing kidney EMT. Sci. Rep..

[54] Tsukui T., Sun K.-H., Wetter J.B., Wilson-Kanamori J.R., Hazelwood L.A., Henderson N.C., Adams T.S., Schupp J.C., Poli S.D., Rosas I.O. (2020). Collagen-producing lung cell atlas identifies multiple subsets with distinct localization and relevance to fibrosis. Nat. Commun..

[55] Ng B., Dong J., Viswanathan S., Widjaja A.A., Paleja B.S., Adami E., Ko N.S.J., Wang M., Lim S., Tan J. (2020). Fibroblast-specific IL11 signaling drives chronic inflammation in murine fibrotic lung disease. FASEB J..

[56] Jiang H., Yang J., Li T., Wang X., Fan Z., Ye Q., Du Y. (2024). JAK/STAT3 signaling in cardiac fibrosis: A promising therapeutic target. Front. Pharmacol..

[57] Tsou P., Palisoc P.J., Ali M., Khanna D., Sawalha A.H. (2021). Genome-Wide Reduction in Chromatin Accessibility and Unique Transcription Factor Footprints in Endothelial Cells and Fibroblasts in Scleroderma Skin. Arthritis Rheumatol..

[58] Aghajanian H., Kimura T., Rurik J.G., Hancock A.S., Leibowitz M.S., Li L., Scholler J., Monslow J., Lo A., Han W. (2019). Targeting cardiac fibrosis with engineered T cells. Nature.

[59] Ma J., Huang Y., Chen J., Li Y., Yao R., Li X., Liang Q., Chen X., Peng C., Liu K. (2025). FAP+ fibroblasts orchestrate tumor microenvironment remodeling in renal cell carcinoma with tumor thrombus. Nat. Commun..

[60] Sigafoos A.N., Paradise B.D., Fernandez-Zapico M.E. (2021). Hedgehog/GLI Signaling Pathway: Transduction, Regulation, and Implications for Disease. Cancers.

[61] Lanzer J.D., Wienecke L.M., Flores R.O.R., Zylla M.M., Kley C., Hartmann N., Sicklinger F., Schultz J.-H., Frey N., Saez-Rodriguez J. (2024). Single-cell transcriptomics reveal distinctive patterns of fibroblast activation in heart failure with preserved ejection fraction. Basic. Res. Cardiol..

[62] Saito S., Kitabatake M., Ouji-Sageshima N., Ogawa T., Oda A., Nishimura T., Nishioka T., Fushimi S., Hara A., Shichino S. (2023). Angiopoietin-like 4 Is a Critical Regulator of Fibroblasts during Pulmonary Fibrosis Development. Am. J. Respir. Cell Mol. Biol..

[63] Ghosh A.K., Vaughan D.E. (2012). PAI-1 in tissue fibrosis. J. Cell Physiol..

[64] Schrempf M., Erl A., Mueller J.C., Hoppmann P., Schömig A., Kastrati A., Koch W. (2010). 4G/5G polymorphism and haplotypes of SERPINE1 in atherosclerotic diseases of coronary arteries. Thromb. Haemost..

[65] Tuleta I., Frangogiannis N.G. (2021). Fibrosis of the diabetic heart: Clinical significance, molecular mechanisms, and therapeutic opportunities. Adv. Drug Deliv. Rev..

[66] Ivey M.J., Kuwabara J.T., Riggsbee K.L., Tallquist M.D. (2019). Platelet-derived growth factor receptor-α is essential for cardiac fibro-blast survival. Am. J. Physiol. Heart Circ. Physiol..

[67] Kanisicak O., Khalil H., Ivey M.J., Karch J., Maliken B.D., Correll R.N., Brody M.J., Lin S.-C.J., Aronow B.J., Tallquist M.D. (2016). Genetic lineage tracing defines myofibroblast origin and function in the injured heart. Nat. Commun..

[68] Kaur H., Takefuji M., Ngai C., Carvalho J., Bayer J., Wietelmann A., Poetsch A., Hoelper S., Conway S.J., Möllmann H. (2016). Targeted Ablation of Periostin-Expressing Activated Fibroblasts Prevents Adverse Cardiac Remodeling in Mice. Circ. Res..

[69] Kuwabara J.T., Hara A., Bhutada S., Gojanovich G.S., Chen J., Hokutan K., Shettigar V., Lee A.Y., DeAngelo L.P., Heckl J.R. (2022). Consequences of PDGFRα(+) fibroblast reduction in adult murine hearts. eLife.

[70] Ubil E., Duan J., Pillai I.C.L., Rosa-Garrido M., Wu Y., Bargiacchi F., Lu Y., Stanbouly S., Huang J., Rojas M. (2014). Mesenchymal–endothelial transition contributes to cardiac neovascularization. Nature.

[71] Mayola M.F., Thackeray J.T. (2023). The Potential of Fibroblast Activation Protein-Targeted Imaging as a Biomarker of Cardiac Remodeling and Injury. Curr. Cardiol. Rep..

[72] Gallant J.P., Hintz H.M., Gunaratne G.S., Breneman M.T., Recchia E.E., West J.L., Ott K.L., Heninger E., Jackson A.E., Luo N.Y. (2024). Mechanistic Characterization of Cancer-associated Fibroblast Depletion via an Antibody-Drug Conjugate Targeting Fibroblast Activation Protein. Cancer Res. Commun..

[73] Jiang Y.-H., Zhou M., Cheng M.-D., Chen S., Guo Y.-Q. (2025). CAR-engineered cytolytic Tregs reverse pulmonary fibrosis and remodel the fibrotic niche with limited CRS. J. Clin. Investig..

[74] Montano-Peguero Y., Verdejo H., Riquelme J., Kogan M.J., Lavandero S. (2025). Nanomedicine for Diagnosis and Treatment of Cardiac Fibrosis. Int. J. Nanomed..

[75] Flugel C.L., Majzner R.G., Krenciute G., Dotti G., Riddell S.R., Wagner D.L., Abou-El-Enein M. (2023). Overcoming on-target, off-tumour toxicity of CAR T cell therapy for solid tumours. Nat. Rev. Clin. Oncol..

[76] Tran E., Chinnasamy D., Yu Z., Morgan R.A., Lee C.-C.R., Restifo N.P., Rosenberg S.A. (2013). Immune targeting of fibroblast activation protein triggers recognition of multipotent bone marrow stromal cells and cachexia. J. Exp. Med..

[77] Li Y., Song D., Mao L., Abraham D.M., Bursac N. (2020). Lack of Thy1 defines a pathogenic fraction of cardiac fibroblasts in heart failure. Biomaterials.

[78] Liu Y., Liu J., Quimbo A., Xia F., Yao J., Clamme J.-P., Zabludoff S., Zhang J., Ying W. (2021). Anti-HSP47 siRNA lipid nanoparticle ND-L02-s0201 reverses interstitial pulmonary fibrosis in preclinical rat models. ERJ Open Res..

[79] Labonia M., Senti M.E., van der Kraak P., Brans M., Dokter I., Streef T., Smits A., Deshantri A., de Jager S., Schiffelers R. (2024). Cardiac delivery of modified mRNA using lipid nanoparticles: Cellular targets and biodistribution after intramyocardial administration. J. Control Release.

[80] Fernandes I., Funakoshi S., Hamidzada H., Epelman S., Keller G. (2023). Modeling cardiac fibroblast heterogeneity from human pluripotent stem cell-derived epicardial cells. Nat. Commun..

[81] Farahani R.M., Xaymardan M. (2015). Platelet-Derived Growth Factor Receptor Alpha as a Marker of Mesenchymal Stem Cells in Development and Stem Cell Biology. Stem. Cells Int..

[82] Basalova N., Alexandrushkina N., Grigorieva O., Kulebyakina M., Efimenko A. (2023). Fibroblast Activation Protein Alpha (FAPα) in Fibrosis: Beyond a Perspective Marker for Activated Stromal Cells?. Biomolecules.

[83] Ieda M., Fu J.-D., Delgado-Olguin P., Vedantham V., Hayashi Y., Bruneau B.G., Srivastava D. (2010). Direct Reprogramming of Fibroblasts into Functional Cardiomyocytes by Defined Factors. Cell.

[84] Ahmad W., Dutta S., He X., Chen S., Saleem M.Z., Wang Y., Liang J. (2025). In Vivo Targeted Reprogramming of Cardiac Fibroblasts for Heart Regeneration: Advances and Therapeutic Potential. Bioengineering.

[85] Ebrahimi B. (2017). In vivo reprogramming for heart regeneration: A glance at efficiency, environmental impacts, challenges and future directions. J. Mol. Cell. Cardiol..

[86] Tani H., Sadahiro T., Yamada Y., Isomi M., Yamakawa H., Fujita R., Abe Y., Akiyama T., Nakano K., Kuze Y. (2023). Direct Reprogramming Improves Cardiac Function and Reverses Fibrosis in Chronic Myocardial Infarction. Circulation.

[87] Qian L., Huang Y., Spencer C.I., Foley A., Vedantham V., Liu L., Conway S.J., Fu J.-D., Srivastava D. (2012). In vivo reprogramming of murine cardiac fibroblasts into induced cardiomyocytes. Nature.

[88] Song K., Nam Y.J., Luo X., Qi X., Tan W., Huang G.N., Acharya A., Smith C.L., Tallquist M.D., Neilson E.G. (2012). Heart repair by reprogramming non-myocytes with cardiac transcription factors. Nature.

[89] Kurotsu S., Sadahiro T., Fujita R., Tani H., Yamakawa H., Tamura F., Isomi M., Kojima H., Yamada Y., Abe Y. (2020). Soft Matrix Promotes Cardiac Reprogramming via Inhibition of YAP/TAZ and Suppression of Fibroblast Signatures. Stem. Cell Rep..

[90] Zhang Z., Zhang W., Blakes R., Sundby L.J., Shi Z., Rockey D.C., Ervasti J.M., Nam Y.L. (2022). Fibroblast fate determination during cardiac reprogramming by remodeling of actin filaments. Stem. Cell Reports.

[91] Zhang Z., Shayani G., Xu Y., Kim A., Hong Y., Feng H., Zhu H. (2023). Induction of Senescence by Loss of Gata4 in Cardiac Fibroblasts. Cells.

[92] Mahmoudi S., Mancini E., Xu L., Moore A., Jahanbani F., Hebestreit K., Srinivasan R., Li X., Devarajan K., Prélot L. (2019). Heterogeneity in old fibroblasts is linked to variability in reprogramming and wound healing. Nature.

[93] Peng W.G., Getachew A., Zhou Y. (2025). Decoding the epigenetic and transcriptional basis of direct cardiac reprogramming. STEM CELLS.

[94] Jayawardena T.M., Egemnazarov B., Finch E.A., Zhang L., Payne J.A., Pandya K., Zhang Z., Rosenberg P., Mirotsou M., Dzau V.J. (2012). MicroRNA-Mediated In Vitro and In Vivo Direct Reprogramming of Cardiac Fibroblasts to Cardiomyocytes. Circ. Res..

[95] Jiang L., Liang J., Huang W., Ma J., Park K.H., Wu Z., Chen P., Zhu H., Ma J.-J., Cai W. (2022). CRISPR activation of endogenous genes reprograms fibroblasts into cardiovascular progenitor cells for myocardial infarction therapy. Mol. Ther..

[96] Fernandez-Perez A., Sathe A.A., Bhakta M., Leggett K., Xing C., Munshi N.V. (2019). Hand2 Selectively Reorganizes Chromatin Accessibility to Induce Pacemaker-like Transcriptional Reprogramming. Cell Rep..

[97] Muraoka N., Yamakawa H., Miyamoto K., Sadahiro T., Umei T., Isomi M., Nakashima H., Akiyama M., Wada R., Inagawa K. (2014). MiR-133 promotes cardiac reprogramming by directly repressing Snai1 and silencing fibroblast signatures. EMBO J..

[98] Garry G.A., Bezprozvannaya S., Chen K., Zhou H., Hashimoto H., Morales M.G., Liu N., Bassel-Duby R., Olson E.N. (2021). The histone reader PHF7 cooperates with the SWI/SNF complex at cardiac super enhancers to promote direct reprogramming. Nat. Cell Biol..

[99] Yamakawa H., Muraoka N., Miyamoto K., Sadahiro T., Isomi M., Haginiwa S., Kojima H., Umei T., Akiyama M., Kuishi Y. (2015). Fibroblast Growth Factors and Vascular Endothelial Growth Factor Promote Cardiac Reprogramming under Defined Conditions. Stem. Cell Rep..

[100] Wang H., Yang J., Cai Y., Zhao Y. (2024). Macrophages suppress cardiac reprogramming of fibroblasts in vivo via IFN-mediated intercellular self-stimulating circuit. Protein. Cell.

[101] Liu Z., Wang L., Welch J.D., Ma H., Zhou Y., Vaseghi H.R., Yu S., Wall J.B., Alimohamadi S., Zheng M. (2017). Single-cell transcriptomics reconstructs fate conversion from fibroblast to cardiomyocyte. Nature.

[102] He X., Liang J., Paul C., Huang W., Dutta S., Wang Y. (2022). Advances in Cellular Reprogramming-Based Approaches for Heart Regenerative Repair. Cells.

[103] Nakano K., Sadahiro T., Fujita R., Isomi M., Abe Y., Yamada Y., Akiyama T., Honda S., French B.A., Mizukami H. (2024). Development of adeno-associated viral vectors targeting cardiac fibroblasts for efficient in vivo cardiac reprogramming. Stem. Cell Rep..

[104] Chaffin M., Papangeli I., Simonson B., Akkad A.-D., Hill M.C., Arduini A., Fleming S.J., Melanson M., Hayat S., Kost-Alimova M. (2022). Single-nucleus profiling of human dilated and hypertrophic cardiomyopathy. Nature.

[105] Liu C., Shen M., Liu Y., Manhas A., Zhao S.R., Zhang M., Belbachir N., Ren L., Zhang J.Z., Caudal A. (2024). CRISPRi/a screens in human iPSC-cardiomyocytes identify glycolytic activation as a druggable target for doxorubicin-induced cardiotoxicity. Cell Stem. Cell.

[106] Zhang H., Thai P.N., Shivnaraine R.V., Ren L., Wu X., Siepe D.H., Liu Y., Tu C., Shin H.S., Caudal A. (2024). Multiscale drug screening for cardiac fibrosis identifies MD2 as a therapeutic target. Cell.

[107] Bernard K., Logsdon N.J., Benavides G.A., Sanders Y., Zhang J., Darley-Usmar V.M., Thannickal V.J. (2018). Glutaminolysis is required for transforming growth factor-β1–induced myofibroblast differentiation and activation. J. Biol. Chem..

[108] Philip J.L., Razzaque M.A., Han M., Li J., Theccanat T., Xu X., Akhter S.A. (2015). Regulation of mitochondrial oxidative stress by β-arrestins in cultured human cardiac fibroblasts. Dis. Model Mech..

[109] Cinato M., Kang R., Kramar S., Savchenko L., Pizzinat N., Swiader A., Kel A., Kalmykov A., Stelmashenko D., Martinelli I. (2025). Transcriptome fingerprinting of aberrant fibroblast activation unlocks effective therapeutics to tackle cardiac fibrosis. Sci. Adv..

[110] Schafer S., Viswanathan S., Widjaja A.A., Lim W.-W., Moreno-Moral A., DeLaughter D.M., Ng B., Patone G., Chow K., Khin E. (2017). IL-11 is a crucial determinant of cardiovascular fibrosis. Nature.

[111] Erasmus M., Samodien E., Lecour S., Cour M., Lorenzo O., Dludla P., Pheiffer C., Johnson R. (2020). Linking LOXL2 to Cardiac Interstitial Fibrosis. Int. J. Mol. Sci..

[112] Sato S., Koyama K., Ogawa H., Murakami K., Imakura T., Yamashita Y., Kagawa K., Kawano H., Hara E., Nishioka Y. (2023). A novel BRD4 degrader, ARV-825, attenuates lung fibrosis through senolysis and antifibrotic effect. Respir. Investig..

[113] Cozzolino C., Floris E., Icolaro F., Pontecorvi V., Bordin A., Frati G., Pagano F., De Falco E., Picchio V., Chimenti I. (2025). Sirtuin-mediated modulation of cardiac fibrosis: Emerging molecular insights and therapeutic perspectives. Pharmacol. Res..

[114] Gan C., Wei W., Xue T., Xie Y., Yue L., Su X., Yu Y., Liu Z., Ye T. (2025). Focal adhesion kinase inhibitors in fibrotic diseases therapy: Development and therapeutic potential. Eur. J. Med. Chem..

[115] Zhang C., Jiao S., Zeng D., Jiang W., Wang R., Zheng B., Wang M., Wang S., Gui X. (2025). IL-11/IL-11R signal inhibition by 9MW3811 remodels immune tumor microenvironment and enhances anti-tumor efficacy of PD-1 blockade. npj Precis. Oncol..

[116] Chimenti I., Pagano F., Cozzolino C., Icolaro F., Floris E., Picchio V. (2025). The Role of Cardiac Fibroblast Heterogeneity in Myocardial Fibrosis and Its Novel Therapeutic Potential. Int. J. Mol. Sci..

[117] Jin Y., Liu Y., Ge A., Yu Y., Wan Y., Li C., Zhang C. (2025). Advances in fibroblast-based cardiac reprogramming in the treatment of heart disease. Inflamm. Res..

[118] He X., Dutta S., Liang J., Paul C., Huang W., Xu M., Chang V., Ao I., Wang Y. (2023). Direct cellular reprogramming techniques for cardiovascular re-generative therapeutics. Can. J. Physiol. Pharmacol..

[119] Inman G.J., Nicolás F.J., Callahan J.F., Harling J.D., Gaster L.M., Reith A.D., Laping N.J., Hill C.S. (2002). SB-431542 Is a Potent and Specific Inhibitor of Transforming Growth Factor-β Superfamily Type I Activin Receptor-Like Kinase (ALK) Receptors ALK4, ALK5, and ALK7. Mol. Pharmacol..

[120] Spender L.C., Ferguson G.J., Hughes G.D., Davies B.R., Goldberg F.W., Herrera B., Taylor R.G., Strathearn L.S., Sansom O.J., Barry S.T. (2019). Preclinical Evaluation of AZ12601011 and AZ12799734, Inhibitors of Transforming Growth Factor β Superfamily Type 1 Receptors. Mol. Pharmacol..

[121] Dubey N., Vishwakarma V.K., Verma A., Goyal A., Yadav H.N., Reeta K.H., Yadav S.C., Parakh N. (2025). Cardiac Fibroblast Receptors: A Preclinical Dive Into Their Regulatory Influence on Cardiovascular Diseases. J. Biochem. Mol. Toxicol..

[122] Tang T.T., Konradi A.W., Feng Y., Peng X., Ma M., Li J., Yu F.X., Guan K.L., Post L. (2021). Small Molecule Inhibitors of TEAD Auto-palmitoylation Selectively Inhibit Proliferation and Tumor Growth of NF2-deficient Mesothelioma. Mol. Cancer Ther..

[123] Barry-Hamilton V., Spangler R., Marshall D., McCauley S., Rodriguez H.M., Oyasu M., Mikels A., Vaysberg M., Ghermazien H., Wai C. (2010). Allosteric inhibition of lysyl oxi-dase–like-2 impedes the development of a pathologic microenvironment. Nat. Med..

[124] Cabrera-Fuentes H.A., Barreto G., Perez-Campos E., Nivon-Torres G.F., González A.A.G., Al-Suhaim E.A., Liehn E.A. (2025). Targeting Inflammation and Fibrosis in Cardiovascular Disease: Emerging Mechanisms and Therapies. FASEB J..

[125] Yang X., Woldemichael K., Guo X., Zhao S., Qian Y., Huang Z.J. (2025). CellREADR: An ADAR-based RNA sensor-actuator device. Methods Enzymol..

